# A dynamic predictive nomogram of long-term survival in primary gastric lymphoma: a retrospective study

**DOI:** 10.1186/s12876-022-02419-2

**Published:** 2022-07-16

**Authors:** Jinru Yang, Tao Liu, Ying Zhu, Fangyuan Zhang, Menglan Zhai, Dejun Zhang, Lei Zhao, Min Jin, Zhenyu Lin, Tao Zhang, Liling Zhang, Dandan Yu

**Affiliations:** Cancer Center, Union Hospital, Tongji Medical College, Huazhong University of Science and Technology, Wuhan, 430022 People’s Republic of China

**Keywords:** Primary gastric lymphoma, Overall survival, Cancer-specific survival, Nomogram, Dynamic prediction

## Abstract

**Background:**

Primary gastric lymphoma (PGL) is the most common extranodal non-Hodgkin lymphoma (NHL). Due to the rarity of the disease, it is important to create a predictive model that provides treatment and prognosis for patients with PGL and physicians.

**Methods:**

A total of 8898 and 127 patients diagnosed with PGL were obtained from the SEER database and from our Cancer Center as training and validation cohorts, respectively. Univariate and multivariate Cox proportional hazards models were used to investigate independent risk factors for the construction of predictive survival nomograms, and a web nomogram was developed for the dynamic prediction of survival of patients with PGL. The concordance index (C-index), calibration plot, and receiver operating characteristics (ROC) curve were used to evaluate and validate the nomogram models.

**Results:**

There were 8898 PGL patients in the SEER cohort, most of whom were married men over the age of 60, 16.1% of the primary tumors were localized in the antrum and pylori of the stomach, which was similar to the composition of 127 patients in the Chinese cohort, making both groups comparable. The Nomogram of overall survival (OS) was compiled based on eight variables, including age at diagnosis, sex, race, marital status, histology, stage, radiotherapy and chemotherapy. Cancer-specific survival (CSS) nomogram was developed with eight variables, including age at diagnosis, sex, marital status, primary tumor site, histology, stage, radiotherapy and chemotherapy. The C-index of OS prediction nomogram was 0.948 (95% CI: 0.901–0.995) in the validation cohort, the calibration plots showed an optimal match and a high area below the ROC curve (AUC) was observed in both training and validation sets. Also, we established the first web-based PGL survival rate calculator (https://yangjinru.shinyapps.io/DynNomapp/).

**Conclusion:**

The web dynamic nomogram provided an insightful and applicable tool for evaluating PGL prognosis in OS and CSS, and can effectively guide individual treatment and monitoring.

**Supplementary Information:**

The online version contains supplementary material available at 10.1186/s12876-022-02419-2.

## Background

Primary gastric lymphoma (PGL) is a malignant extranodal lymphoma originating from gastric submucous lymphoid tissue [1–3]. PGL represents almost 30-40% of extranodal lymphomas and approximately 5% of all gastric malignancies [1–3]. The two predominant histological subtypes are extranodal marginal zone lymphoma of mucosa-associated lymphoid tissue (MALT) and diffuse large B cell lymphoma (DLBCL), constituting around 90% of all cases [1–3]. So far, there is still no consensus on the optimal treatment, especially for *H. pylori* negative MALT, DLBCL, and advanced PGL [4, 5]. Given the rareness of this disease, treatment recommendations are mostly based on case-series data, rather than large randomized clinical trials [6]. The Ann-Arbor stage system is a tool commonly used by oncologists to predict disease progression and design therapeutic strategies for lymphoma, taking the location of lymph node spread as the basis for staging. However, it does not include other factors that may affect survival, such as personal and cancer treatment information [1, 3]. Considering various factors affecting the etiology of lymphoma, the prognosis based on the Ann-Arbor staging system alone is unreliable [7]. Nomogram is a reliable and convenient prognostic tool, which has been widely used to predict the overall probability of specific outcomes in clinical oncology by incorporating a number of prognostic factors [8–13]. It can use known and important prognostic factors to quantitatively predict the prognosis in certain patients and explain the numerical probability of clinical outcomes [8, 9]. A prognostic tool [14] has recently been proposed for primary gastric DLBCL. However, a complete nomogram for predicting survival in patients with PGL has not been reported. The purpose of this study is to develop comprehensive and effective nomogram based on the data retrieved from the SEER and Chinese cohorts to better predict the survival rate of PGL patients.

## Methods

### Study population

The Surveillance, Epidemiology, and End Results (SEER) project sponsored by the National Cancer Institute (NCI) is a free public database (https://seer.cancer.gov/). It consists of 18 registered cancer centers and currently covering approximately 30% of the United States population. We got access to the SEER Research Data. SEER*Stat software (Version 8.3.9.2; NCI; Bethesda, MD) was used to extract clinical data of patients with PGL from the SEER in 2000-2018 (November 2020 Submission). Patients with PGL were identified by the International Classification of Diseases for Oncology, the third edition (ICD-O-3) histologic codes 9590-9599, 9650-9669, 9670-9699, 9700-9719, 9720-9729 for lymphoma and primary site codes C16.0-C16.9 for gastric. Patients with more than one primary tumor site, without pathological confirmation, race records, marital status, stage records, survival months and active follow-up were excluded from the extracted patients. The demographic and clinical characteristics obtained in the database are as follows: age at diagnosis, sex, race, marital status, primary tumor site, histology, cancer stage, surgery therapy, radiotherapy, chemotherapy and survival months until death or last follow-up. The cancer stages were according to the Ann Arbor staging system, which can be found in the American Joint Committee on Cancer (AJCC) Cancer Staging Manual (7th edition) or Union for International Cancer Control (UICC) staging manual. The overall survival (OS) was calculated from the date of diagnosis to the date of death or the date of censoring, regardless of whether the patients survived at the last follow-up. The cancer-specific survival (CSS) was calculated from the diagnosis date to death date only from PGL or censorship date if patients were alive or dead from another cause.

For clinical data, patients initially diagnosed with PGL from May 2015 to September 2021 in our Cancer Center were included in this retrospective study, the last follow-up was in November 2021. The inclusion and exclusion criteria were in line with the SEER cohort above. However, cancer-specific deaths have not been observed in the Chinese cohort. This study was approved by the Ethical Committees of Union Hospital, Tongji Medical College, Huazhong University of Science and Technology and in accordance with the Helsinki Declaration.

### Statistical analysis

In the SEER database, we identified 8898 eligible PGL patients in order to compile the effective OS and CSS nomograms. One hundred and twenty-seven patients initially diagnosed with PGL were assigned to the validation cohort. Cox proportional hazards model was used for univariate and multivariate analyses to identify the variables that significantly influenced OS and CSS in the training cohort. Then, OS and CSS nomograms based on independent prognostic factors were constructed by training cohort. The OS nomogram was validated in both the training and the validation cohort. The predictive performance of nomograms was evaluated with discrimination and calibration tests. Concordance index (C-index) and receiver operating characteristic curve (ROC) were used for assessing the discrimination, and calibration curve was used to compare actual results and survival probability predicted by nomogram. For this purpose, bootstraps with 1000 resamples were used [15]. The web-based OS and CSS probability calculators were built using packages “DynNom” and “shiny” in R software. Statistical analysis was performed using the SPSS Statistics software (version 23.0; IBM Corporation; Armonk, NY) and the R software (version 4.0.3). Hazards ratios and 95% confidence intervals (95% CI) were calculated. Two-sided P value less than 0.05 was considered statistically significant.

## Results

### Demographic and clinical characteristics

A total of 8898 patients in the SEER cohort and 127 patients in the Chinese cohort met the inclusion criteria. Table 1 summarizes the demographic and clinical characteristics of patients in the training and validation cohorts. In SEER training cohort, most patients aged between 60 and 79 years old (48.57%). Over half of eligible patients were male (54.57%) and married (58.65%). The majority of patients were white (80.22%), followed by black (9.53%). The primary site of the patients' tumor was 12.13% in the cardiac and gastric fundus, 13.49% in the gastric body, 16.10% in antrum and pylorus, and 9.59% in the lesser and greater curvature. DLBCL and MALT histologic subtype accounted for 42.23% and 44.83%, respectively. About 61.77% of patients were diagnosed with Ann Arbor phase I disease. Of the PGL patients, 50.24% received chemotherapy, only 10.29% underwent surgery and 24.53% performed radiotherapy.

**Table 1:** Demographic and clinical characteristics in the training and validation cohorts

Characteristic	Training cohort (n = 8898)		Validation cohort n = 127)	
	Number	%	Number	%
*Age*				
<40	404	4.54	13	10.24
40-59	2229	25.05	70	55.12
60-79	4322	48.57	44	34.65
≥80	1943	21.84	0	0
*Sex*				
Male	4856	54.57	52	40.94
Female	4042	45.43	75	59.06
*Race*				
White	7138	80.22	0	0
Black	848	9.53	0	0
Other	912	10.25	127	100
*Marital status*				
Married	5219	58.65	120	94.49
Separated/Divorced	803	9.02	2	1.57
Widowed	1590	17.87	1	0.79
Single	1286	14.45	4	3.15
*Primary tumor sites*				
Cardiac/Fundus	1079	12.13	19	14.96
Body	1200	13.49	48	37.80
Antrum/Pylorus	1433	16.10	47	37.00
Lesser/Greater curvature	853	9.59	3	2.36
Other	4333	48.70	10	7.87
*Histologic subtype*				
DLBCL	3758	42.23	53	41.73
MALT	3989	44.83	55	43.31
Other B cell lymphoma	483	5.43	8	6.30
T/NK cell lymphoma	108	1.21	4	3.15
Other NHL	352	3.96	3	2.36
HL	16	0.18	0	0
NOS	192	2.16	4	3.15
*Stage*				
stage I	5496	61.77	30	23.62
stage II	1363	15.32	22	17.32
stage III	506	5.69	0	0
stage IV	1533	17.23	35	27.56
Unknown	NA	NA	40	31.50
*Surgery*				
Yes	916	10.29	18	14.17
No/Unknown	7982	89.71	109	85.83
*Radiotherapy*				
Yes	2183	24.53	54	42.52
No/Unknown	6715	75.47	73	57.48
*Chemotherapy*				
Yes	4470	50.24	78	61.42
No/Unknown	4428	49.76	49	38.58
*Status*				
Alive	4104	46.12	119	93.70
Dead	4794	53.88	8	6.30
*Cancer-specific dead*				
Yes	2267	25.48	NA	NA
No	6631	74.52	NA	NA

### Identifying independent prognostic factors for OS and CSS

Univariate and multivariate Cox proportional hazards analysis of OS and CSS in patients with PGL in the training cohort were listed in Tables 2 and 3. Univariate analyses showed that age at diagnosis, sex, race, marital status, histology, stage, radiotherapy and chemotherapy were associated with OS. Multivariate analyses identified eight variables, including age at diagnosis, sex, race, marital status, histology, stage, radiotherapy and chemotherapy, to be significantly associated with OS. Age at diagnosis, sex, marital status, primary tumor site, histology, stage, surgery, radiotherapy and chemotherapy were closely related to CSS in univariate analyses. Age at diagnosis, sex, marital status, primary tumor site, histology, stage, radiotherapy and chemotherapy were independent risk factors for CSS in multivariate analyses.

**Table 2:** Cox proportional hazard regression analysis of OS in patients with primary gastric lymphoma in the training cohort (n=8898)

Characteristic	Univariable analysis			Multivariable analysis		
	HR	95% CI	*P* value	HR	95% CI	*P* value
*Age*						
<40	Reference			Reference		
40-59	1.122	0.916–1.375	0.265	1.311	1.067–1.610	**0.010**
60-79	2.419	1.994–2.934	< **0.001**	2.819	2.314–3.433	< **0.001**
≥80	6.081	5.001–7.394	< **0.001**	6.573	5.364–8.053	< **0.001**
*Sex*						
Male	Reference			Reference		
Female	0.889	0.840–0.941	< **0.001**	0.755	0.710–0.803	< **0.001**
*Race*						
White	Reference			Reference		
Black	0.991	0.899–1.092	0.849	1.177	1.065–1.301	**0.001**
Other	0.838	0.759–0.926	**0.001**	0.956	0.864–1.057	0.380
*Marital status*						
Married	Reference			Reference		
Separated/Divorced	1.187	1.072–1.315	**0.001**	1.295	1.168–1.436	< **0.001**
Widowed	2.227	1.055–1.257	< **0.001**	1.585	1.465–1.714	< **0.001**
Single	1.152	1.055–1.257	**0.002**	1.387	1.267–1.518	< **0.001**
*Primary tumor sites*						
Cardiac/Fundus	Reference			Reference		
Body	0.987	0.881–1.105	0.987	–	–	–
Antrum/Pylorus	0.930	0.836–1.035	0.930	–	–	–
Lesser/Greater curvature	1.003	0.889–1.132	0.955	–	–	–
Other	1.058	0.968–1.156	0.212	–	–	–
Histologic subtype	48					
DLBCL	Reference			Reference		
MALT	0.529	0.497–0.563	< **0.001**	0.534	0.495–0.575	< **0.001**
Other B cell lymphoma	1.001	0.889–1.128	0.986	0.888	0.786–1.003	0.055
T/NK cell lymphoma	1.721	1.389–2.132	< **0.001**	1.876	1.511–2.329	< **0.001**
Other NHL	1.017	0.886–1.168	0.810	0.977	0.850–1.122	0.738
HL	1.351	0.766–2.383	0.298	1.172	0.663–2.072	0.584
NOS	1.050	0.883–1.249	0.582	0.975	0.818–1.162	0.777
*Stage*						
stage I	Reference			Reference		
stage II	1.241	1.144–1.347	< **0.001**	1.244	1.143–1.354	< **0.001**
stage III	1.610	1.435–1.807	< **0.001**	1.484	1.316–1.672	< **0.001**
stage IV	1.850	1.722–1.988	< **0.001**	1.742	1.611–1.884	< **0.001**
*Surgery*						
Yes	Reference			Reference		
No/Unknown	0.934	0.854–1.021	0.131	–	–	–
*Radiotherapy*						
Yes	Reference			Reference		
No/Unknown	1.632	1.518–1.755	< **0.001**	1.297	1.204–1.396	< **0.001**
*Chemotherapy*						
Yes	Reference			Reference		
No/Unknown	0.860	0.813–0.911	< **0.001**	1.310	1.221–1.405	< **0.001**

The bold numbers indicate that the variables have statistical differences (*P* < 0.05)

**Table 3:** Cox proportional hazard regression analysis of CSS in patients with primary gastric lymphoma in the training cohort (n=8898)

Characteristic	Univariable analysis			Multivariable analysis		
	HR	95% CI	*P* value	HR	95% CI	*P* value
*Age*						
<40	Reference			Reference		
40–59	0.812	0.642–1.027	0.082	1.016	0.801–1.290	0.895
60–79	1.271	1.019–1.586	**0.033**	1.617	1.287–2.033	< **0.001**
≥80	2.580	2.059–3.232	**<0.001**	3.000	2.361–3.813	< **0.001**
*Sex*						
Male	Reference			Reference		
Female	0.880	0.810–0.957	**0.003**	0.816	0.746–0.892	< **0.001**
*Race*						
White	Reference			Reference		
Black	1.137	0.993–1.302	0.063	–	–	–
Other	1.059	0.926–1.211	0.404	–	–	–
*Marital status*						
Married	Reference			Reference		
Separated/Divorced	1.174	1.010–1.364	**0.037**	1.254	1.078–1.458	**0.003**
Widowed	1.960	1.771–2.169	< **0.001**	1.581	1.407–1.776	< **0.001**
Single	1.360	1.207–1.531	< **0.001**	1.483	1.311–1.677	< **0.001**
*Primary tumor sites*						
Cardiac/Fundus	Reference			Reference		
Body	0.999	0.843–1.184	0.991	1.171	0.986–1.389	0.072
Antrum/Pylorus	0.994	0.804–1.108	0.481	1.022	0.870–1.200	0.793
Lesser/Greater curvature	0.897	0.744–1.082	0.255	0.938	0.778–1.132	0.508
Other	1.211	1.062–1.380	**0.004**	1.185	1.040–1.352	**0.011**
*Histologic subtype*						
DLBCL	Reference			Reference		
MALT	0.238	0.214–0.265	< **0.001**	0.248	0.219–0.281	< **0.001**
Other B cell lymphoma	1.047	0.900–1.218	1.047	0.848	0.727–0.990	**0.036**
T/NK cell lymphoma	1.790	1.379–2.323	< **0.001**	1.762	1.355–2.291	< **0.001**
Other NHL	0.932	0.775–1.121	0.455	0.867	0.720–1.045	0.134
HL	1.516	0.757–3.038	0.240	1.198	0.595–2.410	0.613
NOS	0.844	0.655–1.086	0.187	0.774	0.599–0.999	**0.049**
*Stage*						
stage I	Reference			Reference		
stage II	1.845	1.643–2.072	< **0.001**	1.588	1.407–1.793	< **0.001**
stage III	2.548	2.183–2.974	< **0.001**	1.991	1.695–2.339	< **0.001**
stage IV	3.305	2.999–3.644	< **0.001**	2.551	2.294–2.838	< **0.001**
*Surgery*						
Yes	Reference			Reference		
No/Unknown	0.866	0.762–0.985	**0.028**	1.063	0.931–1.214	0.368
*Radiotherapy*						
Yes	Reference			Reference		
No/Unknown	1.833	1.642–2.047	< **0.001**	1.209	1.079–1.354	**0.001**
*Chemotherapy*						
Yes	Reference			Reference		
No/Unknown	0.608	0.559–0.662	< **0.001**	1.491	1.346–1.652	< **0.001**

The bold numbers indicate that the variables have statistical differences (*P* < 0.05)

### Nomogram construction

**Fig. 1:**
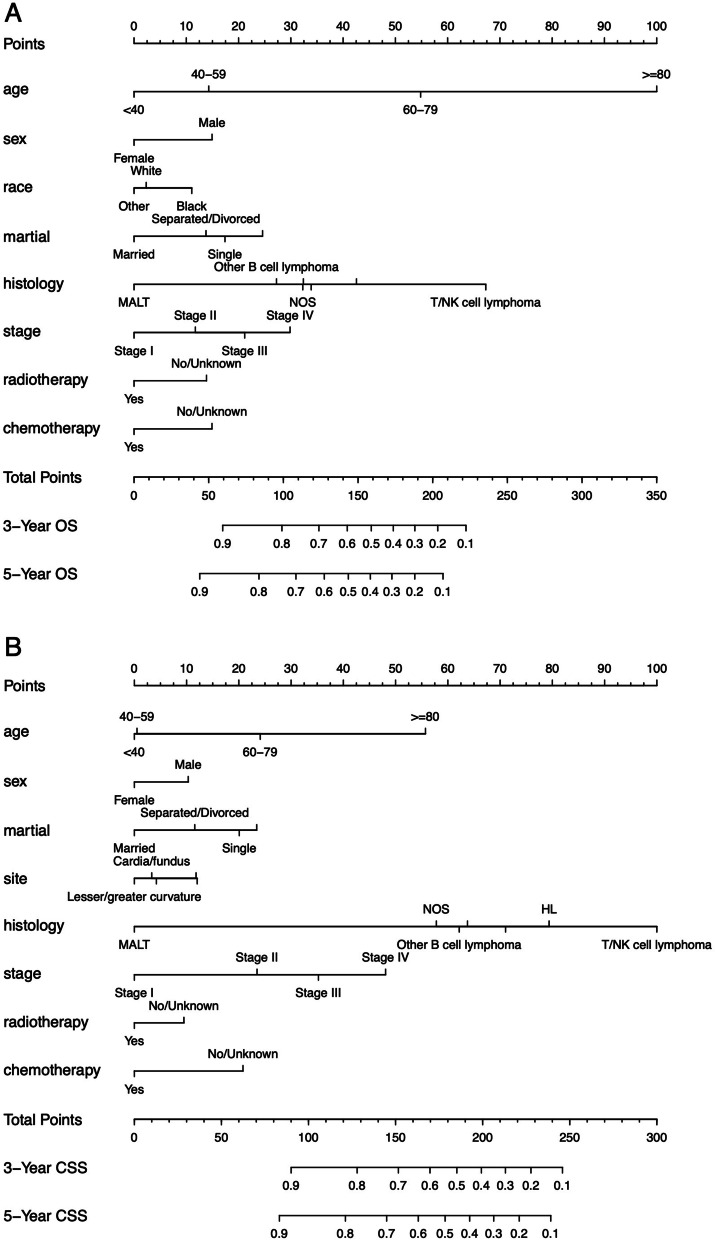
OS and CSS associated nomograms for PGL patients. **A** OS nomograms for PGL in 3-year and 5-year; **B** CSS nomograms for PGL in 3-year and 5-year.

The prognostic nomograms based on sorted significant independent factors from multivariate analysis for predicting 3-year or 5-year OS and CSS in the training cohort were shown in Fig. 1. Scores were given at each level of each variable, and the total score was obtained by adding a score on the point scale of each selected variable, which subsequently helped correlate with the probability of the event for each patient.

### Nomogram validation

**Fig. 2:**
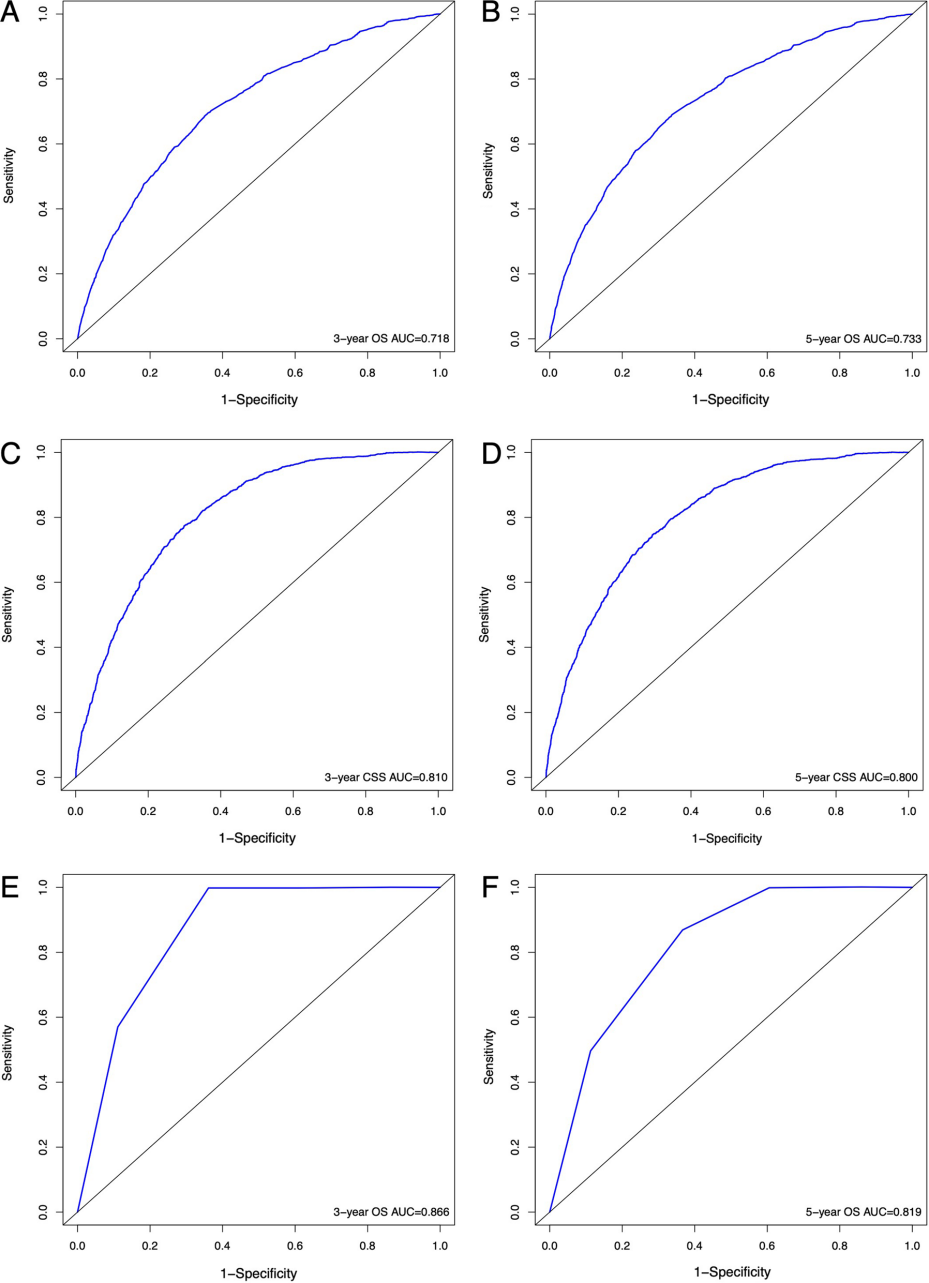
ROC curves for the nomograms. The ROC curve for the nomogram with 3-year OS **A** and 3-year CSS **C** in the training cohort and 3-year OS **E** in the validation cohort; with 5-year OS **B** and 5-year CSS **D** in the training cohort and 5-year OS **F** in the validation cohort.

**Fig. 3:**
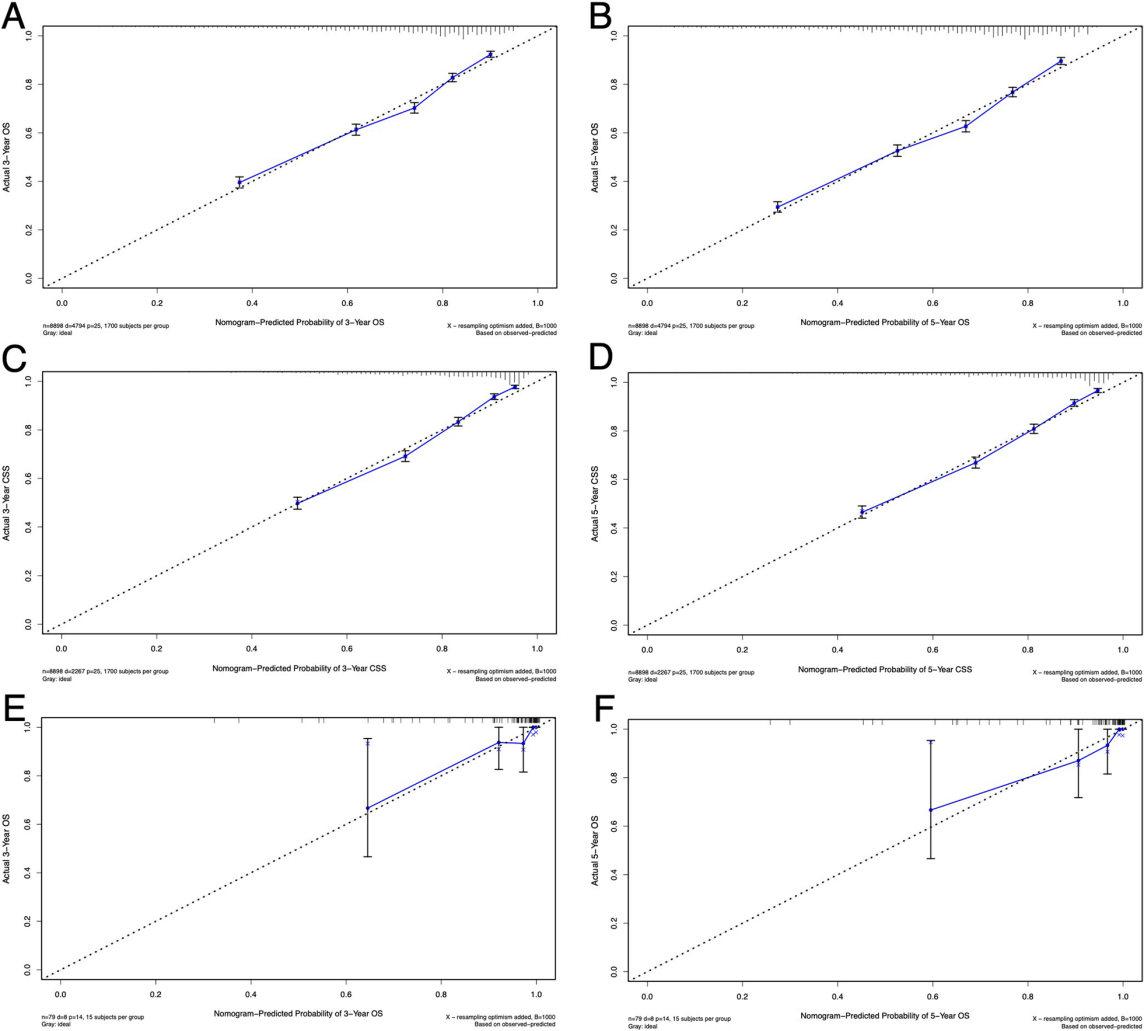
Calibration plots for the nomograms. The calibration plots for the nomogram with 3-year OS **A** and 3-year CSS **C** in the training cohort and 3-year OS **E** in the validation cohort; with 5-year OS **B** and 5-year CSS **D** in the training cohort and 5-year OS **F** in the validation cohort.

The OS nomograms were validated externally. In the training cohort, the prognostic nomogram C-index predictied by OS and CSS was 0.716 (95% CI: 0.708–0.794) and 0.767 (95% CI: 0.757–0.777), respectively. While, in the validation cohort, the C-index was 0.948 (95% CI: 0.901–0.995). However, due to the lack of cancer-specific survival information, it was not possible to obtain the C-index of the validation cohort. The calibration plots of the training and validation cohorts for 3-year or 5-year OS showed an optimal match between nomogram prediction and actual observed outcomes (Fig. 2). A high area below the ROC curve (AUC) was also observed in both training and validation sets, respectively (Fig. 3).

### Web-based probability calculators

**Fig. 4:**
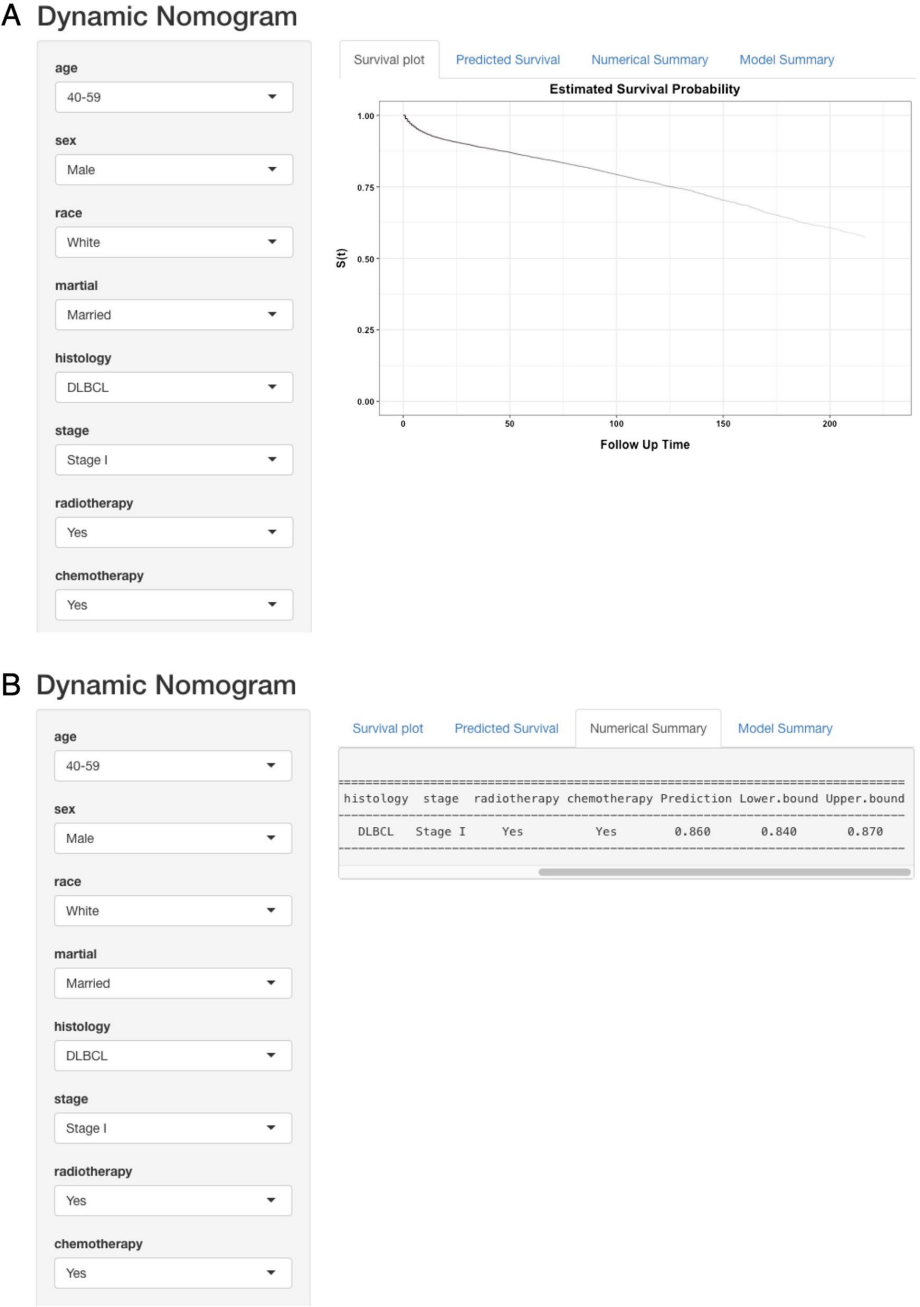
A web-based PGL probability calculator. The 5-year survival probability of PGL with DLBCL, 40–59 years old, male, white, married, stage I patients with radiotherapy, chemotherapy and without surgery showed in the dynamical nomogram. **A** The estimated survival probability. **B** Numerical summary showed the probability and its 95% CI.

According to the multivariate results above, a dynamic nomogram was created for prediction of OS probability in patients with PGL, which was of great convenient and intuitive to individually prognosis prediction based on the personal characteristics of PGL patients (https://yangjinru.shinyapps.io/DynNomapp/). For instance, the 5-year survival rate of DLBCL patients was approximately 86.0% (95% CI: 84.0%-87.0%) in 40-59 years old, male, white, married, stage I patients with radiotherapy, chemotherapy and without surgery, see details in Fig. 4, and in patients with MALT more than 80 years old, female, black, separated/divorced, stage IV, with chemotherapy, without surgery and radiotherapy, 33.0% (95% CI: 27.6–40.0%) of them would survive within 60 months (Additional File 1: Fig. S1).

## Discussion

PGL is the most common extranodal NHL and ranks as the second most common tumour of the stomach [1–3]. PGL is a relatively rare cancer and is easily misdiagnosed due to its non-specific symptoms [16–18]. There are many treatment options include gastrectomy, radiotherapy, chemotherapy, immunotherapy, and observation, while gastrectomy remains controversial due to a considerably favorable prognosis versus quality of life [19–25]. A previous study by Wang et al [2] showed that advanced stage and malignant pathological type are significantly associated with poor overall survival. Most studies demonstrated that female gender, low-grade histology, good PS, and surgical resection were associated with better overall survival [2, 18, 21]. Our study corresponds to these previous reports except for gastrectomy. The balance between efficacy of gastrectomy and quality of life requires further studies.

Although the Ann-Arbor staging system is widely used and recognized for PGL forecasting, it neglects some significant risk factors such as age, race and marital status [26, 27]. In addition, the International Prognostic Index (IPI) and related indices can also divide the prognosis of lymphoma into risk groups. The evaluation criteria included age, stage, ECOG score, extranodal lesions and LDH level, but do not include cancer-related treatment information, therefore it is necessary to develop a more systematic model for predicting PGL risk and to make better therapeutic strategies for individual patients. It is known that nomograms were used to predict the survival status of various diseases [28]. Therefore, we constructed a comprehensive nomogram based on different risk factors to better predict the prognosis of PGL patients. Although the clinical treatment of inert lymphoma, such as MALT and invasive lymphoma is different, the nomogram can well distinguish PGL by pathological type, and better guide the treatment and predict prognosis. These nomograms were able to perform more accurate evaluation and predictions in patients with PGL in both training and validation cohorts, and the results of C-index and calibration curves showed that the models were repeatable and reliable. As far as we know, this is a comprehensive and in-depth large population study aimed at building nomograms for PGL patients, and a web-based dynamic nomogram can directly help clinicians quantify the probability to provide personalized and accurate treatment for patients and to determine the best follow-up time according to disease progression and recurrence rate. CSS is an epidemiological statistical method that can rule out non-cancer deaths. It is an ideal prediction model, which differs from the actual situation, but the index can better predict the mortality attributable to the cancer, and better design precise treatment strategies for patients.

However, there are several limitations in our study. First, our nomogram has been externally validated in a single cancer center, which requires multi-center and large samples verification. Second, some potentially vital information related to the prognosis, such as surgical details, the surgical margin status, vascular invasion, hematological indicators, molecular pathologic characteristics, cell of origin (COO), immunohistochemical results and *Helicobacter pylori* infection status, family history were not included in the SEER database, which could improve predictive ability if incorporated. Third, the majority race of SEER cohort was white and all patients in the Chinese cohort were yellow, therefore the study could be with potential racial heterogeneity. Finally, due to lack of data, we excluded patients from the study, which may lead to potential selection bias.

## Conclusion

To sum up, we constructed a web dynamic nomogram based on the SEER database to better predict the prognosis of PGL patients, and pre-validated it in the Chinese cohort. The nomogram only needs basic information, and has a wider range of application.

## Supplementary Information

**Additional file 1: Figure S1**. A web-based PGL probability calculator. The 5-year survival probability of PGL with MALT, ≥80 years old, female, black, separated/divorced, stage IV patients with chemotherapy, without surgery and radiotherapy showed in the dynamical nomogram. **A** The estimated survival probability. **B** Numerical summary showed the probability and its 95% CI.

## Data Availability

The datasets generated and analysed during the current study are available in the SEER research database (https://seer.cancer.gov/). SEER*Stat software (Version 8.3.9.2; NCI; Bethesda, MD) was used to extract clinical data for patients with PGL from the SEER for the period 2000–2018 (November 2020 Submission).

## Abbreviations

GC: gastric cancer
PGL: primary gastric lymphoma
C-index: concordance index
ROC: receiver operating characteristics
OS: overall survival
CSS: cancer-specific survival
SEER: The surveillance, epidemiology, and end results
HL: Hodgkin lymphoma
NA: Not available
