# Supplementary figures and images for: A dynamic predictive nomogram of long-term survival in primary gastric lymphoma: a retrospective study
Source: BMC Gastroenterol. 2022 Jul 16;22:347. doi: 10.1186/s12876-022-02419-2 (PMC9288002; doi:10.1186/s12876-022-02419-2)

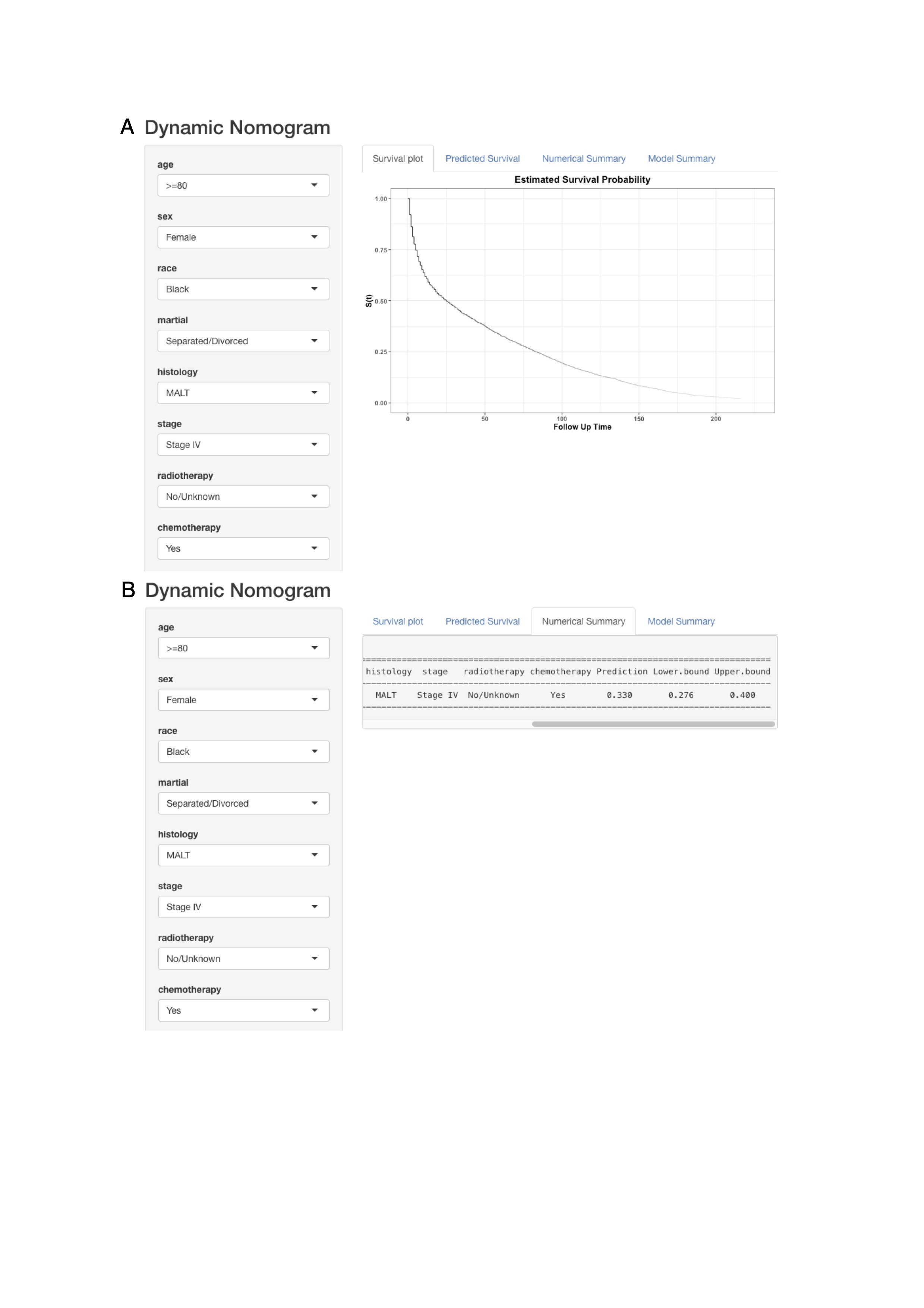

Supplement: Supplementary file 1 — Additional file 1: Figure S1. A web-based PGL probability calculator. The 5-year survival probability of PGL with MALT, ≥80 years old, female, black, separated/divorced, stage IV patients with chemotherapy, without surgery and radiotherapy showed in the dynamical nomogram. A The estimated survival probability. B Numerical summary showed the probability and its 95% CI. [file 12876_2022_2419_MOESM1_ESM.jpg]
